# Cognitive impairment, frailty and rehabilitation outcome in older cardiorespiratory patients. DEC_FRAinRIAB: Study protocol

**DOI:** 10.1371/journal.pone.0272132

**Published:** 2022-08-04

**Authors:** Martina Vigorè, Nicolò Granata, Simona Sarzi Braga, Giancarlo Piaggi, Silvia Audifreddi, Marina Ferrari, Maria Teresa La Rovere, Antonia Pierobon

**Affiliations:** 1 Istituti Clinici Scientifici Maugeri, IRCCS, Psychology Unit of Montescano Institute, Pavia, Italy; 2 Istituti Clinici Scientifici Maugeri, IRCCS, Cardiac Rehabilitation Division of Tradate Institute, Varese, Italy; 3 Istituti Clinici Scientifici Maugeri, IRCCS, Respiratory Rehabilitation Division of Montescano Institute, Pavia, Italy; 4 Istituti Clinici Scientifici Maugeri, IRCCS, Health Direction of Tradate Institute, Varese, Italy; 5 Istituti Clinici Scientifici Maugeri, IRCCS, Cardiac Rehabilitation Division of Montescano Institute, Pavia, Italy; Kurume University School of Medicine, JAPAN

## Abstract

**Background:**

Chronic heart failure (CHF) and chronic obstructive pulmonary disease (COPD) are two clinical conditions often associated with functional worsening, cognitive dysfunctions, treatment non-adherence, psychological distress and poor quality of life (QoL). In addition, since patients suffering from these conditions are often older adults, the presence of frailty syndrome could worsen the clinical situation.

**Methods and design:**

This study protocol of a prospective multi-center clinical trial, will be conducted at two hospitals of the Istituti Clinici Scientifici Maugeri IRCCS group, from July 2020 until December 2022. CHF and COPD older patients (age ≥65) will undergo a multidisciplinary assessment at admission, discharge and at 6 months follow-up, from an inpatient rehabilitation program: disease-related clinical characteristics, functional variables, cognitive screening, treatment adherence, anxiety, depression, QoL and frailty. The estimated sample size will consist of 300 patients

**Discussion:**

The expected results are related to the possibility of an improvement in the areas of intervention after the rehabilitative program and the maintenance of these improvements over time. The assessment of clinical and functional status, cognitive impairment, treatment adherence, psychosocial characteristics, and frailty could provide more specific and useful information about the main features to be considered in the evaluation and treatment of older patients suffering from CHF and COPD undergoing a rehabilitative pathway.

**Trial registration:**

The study has been registered on January 28, 2022 with the ClinicalTrials.gov NCT05230927 registration number (clinicaltrials.gov/ct2/show/NCT05230927).

## Introduction

Chronic heart failure (CHF) and chronic obstructive pulmonary disease (COPD) are global epidemics with a substantial burden on morbidity and mortality [1, 2].

Specifically, CHF is the inability of the heart to pump a sufficient amount of blood through the cardiovascular system. This condition is typically caused by either reduced contractility and/or reduced filling of the left ventricle. CHF prevalence varies widely ranging from 1 to 12% of people in the world and it increases with age: from around 1% for people aged <55 years to 10% in those aged 70 years or older. Furthermore, it is a chronic condition associated with high rates of hospitalization, re-admissions, severe disability, and a high risk of mortality [2]. Concerning COPD, it is a lung disease characterized by a partially irreversible chronic obstruction of lung airflow, with a typical onset after 55 years old [1, 3]. In 2010, it was estimated that the number of COPD cases reached 384 million, with a global prevalence of 11.7% [95% confidence interval (CI) 8.4%-15.0%], and mortality rates are considered to be around 3 million people per year [1]. Both clinical conditions present manifold challenges to healthcare providers, in particular, due to the multiple interactions between each other. For instance, COPD is often responsible for delayed diagnosis of CHF and vice versa, since both clinical conditions have similar symptoms such as dyspnea and poor exercise tolerance [4].

A consistent number of studies in the last few years highlighted that the functional and clinical worsening in patients suffering from cardiac and/or respiratory disease/s increases the risk of cognitive decline. This impairment mainly affects the frontal lobe and subcortical areas of the brain, leading to various deficits concerning executive functions, attention, memory (working memory and learning abilities), and psychomotor speed [5–12].

Moreover, the progressive aging of the population and the improvement of surgical and therapeutic intervention techniques raised the number of older patients suffering from heart disease, severe respiratory disease and/or from different comorbid chronic diseases. In this multifaceted framework of clinical situations, the complex management of therapies by patients led to an important risk that is the non-adherence to therapeutic prescriptions. The latter has serious consequences in terms of disability, hospitalizations, re-hospitalizations and a higher mortality rate [13]. In chronic disease, emotional factors, such as anxiety and depression, also play an important role in disease adaptation and in the rehabilitation outcome in both cardiac [14, 15] and respiratory diseases [14, 16, 17]. Moreover, the health-related quality of life (HRQoL) resulted to be low in these patients, and it appeared to decrease with the worsening of the clinical, cognitive, and/or functional condition [18, 19].

Besides cognitive decline, emotional and adherence issues, the frailty syndrome is a relevant comorbidity within cardiac and respiratory diseases, in particular in the older adult population. Frailty is associated with the loss of functionality that leads to greater vulnerability to adverse events such as the increased risk of falls, hospitalization, institutionalization, disability, and mortality [20].

Frailty screening or assessment scales provide predictive information on the risk of death and institutionalization timing, and their score represent a good predictor of acute clinical condition outcomes too [21, 22]. Specifically, in COPD patients, frailty results a reliable outcome measure, an independent predictor of rehabilitation programs interruption and it was described as a reversible condition in the short term after rehabilitation [23–26]. However, to the best of our knowledge, frailty is still poorly considered and measured in cardiorespiratory rehabilitation settings [27]. Considering the aging population and the promising prognostic use of this index, its assessment can be particularly crucial for maximizing the clinical outcome.

In this vein, the current study aims to bridge this gap. In particular, this paper presents the study protocol of a prospective clinical trial that evaluates and compares socio-demographic, clinical and functional characteristics, cognitive impairment, treatment adherence, anxiety, depression, perceived QoL, and frailty in a sample of older (age ≥65) CHF and/or COPD inpatients, at admission, discharge, and after 6 months from a cardiac or pulmonary rehabilitation program. Another aim is to verify the appropriateness of a frailty screening tool, the Clinical Frailty Scale (CFS), in cardiorespiratory rehabilitation setting, by comparing it with the gold standard, Frailty Index.

## Methods/design

### Participants

All inpatients admitted to the Cardiac Rehabilitation Department and the Pulmonary Rehabilitation Department of ICS Maugeri–Tradate (Varese) and Montescano (Pavia) were evaluated for their eligibility in the study described below.

In particular, CHF is defined as I) signs (e.g. elevated jugular venous pressure, pulmonary crackles, and peripheral edema) and symptoms of HF [New York Heart Association (NYHA) functional class II-IV] in the presence of reduced ejection fraction (LVEF <40%); or II) signs and symptoms of HF (e.g. elevated brain natriuretic peptides and significant structural heart disease/diastolic dysfunction) with mid-range ejection fraction (LVEF 40–49%) or preserved (LVEF ≥50%) [2].

Furthermore, COPD is defined according to the Global initiative for chronic Obstructive Lung Disease (GOLD) criteria (Stage II-IV, C–D). Patients should be in a clinically stable condition (no exacerbations in the last 3 months) with optimized pharmacological therapy (inhalation therapy with long-acting anticholinergic and/or β2-agonists, inhaled corticosteroids when needed) [1].

The exclusion criteria were severe clinical (chronic inflammatory diseases, neoplasia) or psychiatric and neurological (at anamnestic or actual clinical evaluation) conditions, no Italian education, illiteracy or relapse into illiteracy, severe visuo-perceptive deficits, refusal to participate in the study or withdrawal during the evaluation, and severe cognitive deterioration (Mini-Mental State Examination–MMSE score <18.3) [28].

### Materials

#### Clinical evaluation

Blood tests for both clinical conditions: complete blood count (CBC), creatinine, azotaemia, sodium, potassium, thyroid stimulating hormone (TSH), glutamic-oxaloacetic transaminase (GOT), glutamic pyrudic transaminase (GPT), bilirubin, vitamin B12, folate, vitamin D and prealbumin.

CHF related clinical values: NYHA class, brain natriuretic peptide (BNP) or N-terminal pro-brain natriuretic peptide (NT-pro-BNP), LVEF, end-diastolic volume (EDD), tricuspid annular plane systolic excursion (TAPSE), and pulmonary artery pressure (PAP).

COPD related clinical values: GOLD class, partial pressure oxygen (PAO2), partial pressure of arterial carbon dioxide (PACO2), exhaled breath condensate pH (EBC pH), forced expiratory volume in the first second (FEV1), forced vital capacity (VC), peripheral oxygen saturation.

#### Functional evaluation

Barthel Index (BI) is a tool to assess functional status and ability to perform daily life activities in patients with different diseases. It is a 10-item scale, which included the following parts: feeding, bathing, grooming, dressing, bowels and bladder, toilet use, transfers, mobility, and climbing stairs. The score ranges from 0 (total dependence) to 100 (complete independence). Each item can be scored with 0, 5, 10, and 15 points [29].

Barthel Index–Dyspnea (BI-D) is a tool for measuring the level of dyspnea perceived in performing basic daily living activities. It is a further evaluation of patients suffering from respiratory diseases developed after and based on the Barthel Index because the latter was principally focused on neurological patients [30].

Braden Scale is a scale composed of six subscales which measure elements of risk that contribute to both higher intensity and duration of pressure, or lower tissue tolerance for pressure. These are: sensory perception, moisture, activity, mobility, friction, and shear. Each item is scored between 1 and 4,with each score accompanied by a descriptor. The lower the score, the greater the risk [31].

Cumulative Illness Rating Scale (CIRS) is one of the few standardized instruments for obtaining physicians’ ratings of health status along a number of different dimensions. This measure calls for clinical ratings of the degree of pathology and impairment in each of 12 major organ groups and in the psychiatric and behavioral category, as well [32].

Short Physical Performance Battery (SPPB) [33] is used to investigate the association between the physical performance and a self-assessed disability by the subjects recruited at a 6-year follow-up. It is also considered an indicator of functional frailty [34]. SPPB evaluates the functional capacity of the lower limbs and it consists of three different tests which have been assigned a partial score ranging from 0 to 4.

Timed Up and Go Test (TUG), is a simple test to measure a person’s mobility level and requires static and dynamic balancing skills. It measures the time it takes a person to get up from a chair, walk ten feet, turn around, come back to the chair and sit down again. During the test, the person should wear shoes and use any mobility aids used in daily living [35].

6-minute walking test (6MWT) is a self-limited test used to measure functional exercise abilities in people with HF, Acute Coronary Syndrome, and COPD. It is a test in which the person is asked to walk as fast as possible compatible with his clinical condition for a time of 6 minutes, measuring the meters traveled [36].

Malnutrition Universal Screening Tool (MUST) is a tool developed by the British Association for Parenteral and Enteral nutrition (BAPEN). It classifies patients as either at low, medium or high malnutrition risk based on an older person’s BMI, history of unintentional weight loss, and the probability of future weight loss based on acute disease [37].

Mini Nutritional Assessment (MNA) is an eighteen question nutritional assessment instrument specifically developed for use in older people. It is comprised of four components: anthropometry (BMI, calf circumference (CC) and mid-arm circumference (MAC) measurement); self-reported health; dietary questions (including weight loss) and clinical health. The MNA is scored out of 30, with scores < 17/30 classified as ‘malnourished’, scores 17–23.5 as ‘at risk of malnourishment’, and scores > 23.5 as ‘well nourished’ [38].

#### Neuropsychological evaluation

Addenbrooke’s Cognitive Examination III (ACE III) is a 20 minutes screening test, designed for the early detection of cognitive deterioration. It provides a score from 0 to 100 and it analyzes the following five cognitive domains: Attention-Orientation (ACE III-AO = 0–18), Memory (ACE III-M 0 0–26), Verbal Fluency (ACE III-VF = 0–14), Language (ACE III-L = 0–26), and Visuospatial abilities (ACE III-VS = 0–16) [39].

Frontal Assessment Battery (FAB) is a neuropsychological screening test used to assess executive functions. It is a quick and easy test to administer, its score ranges from 0 to 18, and it is divided into the following six sub-tests: conceptualization, mental flexibility, motor programming, sensitivity to interference, inhibitory control, and environmental dependency [40].

In this research, ACE III scores allow to divide the sample into two subpopulations: patients affected by MCI (ACE III-total impaired ≤ 68.68 or borderline = 68.69–75.93 score, or impaired score in at least one subtest: ACE III-AO≤13.2, ACE III-M≤13.04, ACE III-VF≤5.52, ACE III-L≤18.39, and ACE III-VS≤9.97) and patients without cognitive impairment. Furthermore, FAB impaired score of ≤ 12.03 detect patients with executive dysfunctions.

#### Treatment adherence

The Antecedents and Self-efficacy on Adherence Schedule (ASonA) is a self- report schedule aimed to evaluate cognitive, behavioral, and emotional determinants of pharmacological and non-pharmacological adherence. It is a 21-item schedule scored on a 5-point Likert scale (0 = not at all, 4 = very much). The schedule comprises 3 subscales: Antecedents (ASonA-A), which include health condition and health-related limitations acceptance, social support and knowledge about health condition; Self- efficacy (ASonA-SE), exploring patients’ self-care strategies, and patients’ ability to adhere to medication assumption and to non-pharmacological recommendations (i.e., physical activity, diet, alcohol consumption, and smoking avoidance); and Affectivity (ASonA-Aff) which measures patients’ emotional aspects in relation to the health condition. In previous studies, similar schedules, belonging to the schedules group of ASonA, revealed to be sensible instruments showing strong correlations between antecedents and self- efficacy in relation to adherence [13].

#### Psychological evaluation and health-related quality of life

Generalized Anxiety Disorder-7 (GAD) is a questionnaire built to measure the severity of anxiety symptoms in the previous two weeks. The questionnaire consists of 7 items and it organizes the severity of the responses on a 4-point Likert scale. The range scores range from 0 to 21, where scores of 5, 10, and 15 are considered cut-offs for mild, moderate, and severe anxiety, respectively [41, 42].

Patient Health Questionnaire-9 (PHQ-9) is a scale used to determine the diagnosis, severity, and subsequent monitoring of depressive illnesses of the patient. It is divided into nine sub-items, to identify depressive symptoms within the last two weeks (following DSM criteria). The final score has a range between 0–27 and it is divided into clinical variability ranges according to a continuum of symptom severity, where scores of 5, 10, 14, and 19 are considered cut-offs for subthreshold, mild major, moderate major, and severe major depression [42, 43].

Patient Health Questionnaire-4 (PHQ-4) is a composite four-item scale created by using the first two items of the GAD-7 and PHQ-9. As with the parent scales, the PHQ–4 responses are scored as 0 (“not at all”), 1 (“several days”), 2 (“more than half the days”), or 3 (“nearly every day”). The total score ranges from 0 to 12 [44].

The EuroQoL 5D-5L (EQ-5D-5L) is a generic quality of life measurement tool. It investigates the following five dimensions: mobility, self-care, usual activities, pain/discomfort, and anxiety/depression. Each item has five levels: 1, no problem; 2, slight problem; 3 moderate problem; 4 severe problem; 5 extreme problem [45].

The EuroQoL Visual Analogue Scale (EQ VAS) consists of a visual analogue scale from 0 (Worst imaginable health state) to 100 (Best imaginable health state), where the subject is asked to indicate the level of self-perceived wellness [45].

#### Frailty evaluation

Clinical Frailty Scale (CFS) is a scale developed in Canada, which is based on clinical judgment only with a score ranging from 1 to 9: 1) Very Fit; 2) Well; 3) Managing Well; 4) Vulnerable; 5) Mildly Frail; 6) Moderately; Frail; 7) Severely frail; 8) Very severely Frail; 9) Terminally Ill [46]. This scale considers clinical data on the subject’s cognition, mobility, functional abilities, and comorbidity, which can be collected through the medical history obtained from the patient, the caregiver, and/or other healthcare providers [46].

Frailty Index (FI) is a frailty measurement tool created through a geriatric assessment study including non-institutionalized patients. The variables taken into consideration are mobility, muscle strength, comorbidities, cognitive deficits, mood, anthropometric indices, mini nutritional assessment, and social support. It is made up of 40 items and 17 additional items relating to the Social Support Scale [22, 47].

### Intervention

#### Physician

The physician is the main coordinator of the multidisciplinary team and starts the individualized rehabilitation project, in which nurse and physiotherapist are always included, while dietician and psychologist intervene when clinically necessary or in research protocol such in this study [14]. The intervention begins with the evaluation of the patient’s clinical status through the disease-related indexes and laboratory parameters. During the rehabilitative pathway the physician provides all the information and advices related to the disease, monitors the clinical condition, promotes healthy behaviours and, optimizes the pharmacological therapy. Before discharge, the physician re-assess the clinical status and provides the medical report with all the evaluations and interventions performed by the multidisciplinary team.

#### Nurse

The nurse is generally the first healthcare operator who meets the patient and has contact with the family members. The nurse assessment includes, at admission and at discharge, the use of validated and sensitive scales (BI, BI-D, and Braden) to verify if the goals of the rehabilitation program have been achieved. The tasks of nurses are to identify the risk factors and intervene in them and in the particular disabilities, and to promote adherence to pharmacological and non-pharmacological therapy.

#### Physiotherapist

The physiotherapist’s intervention aims to achieve the patient’s maximum possible recovery of functional capacity and autonomy. Treatment consists in two daily sessions lasting from 30 to 45 minutes of cycle-ergometer and/or treadmill, leg ergometer and breathing exercises. The initial assessment phase consisting of SPPB, TUG, and 6MWT allows the selection of the most appropriate treatment related to the patient’s functional needs, while the final assessment permits to verify the results achieved and to plan an appropriate maintenance program.

#### Dietician

Nutritional interventions are focused on the evaluation of the risk of malnutrition through specific assessment (MUST, MNA), on the modification of unhealthy eating habits and on the treatment of obesity, dyslipidemia, hypertension and diabetes mellitus. The nutritional information are based on the evidence in favor of adopting the Mediterranean diet. The dietician adopts the nutrition care process methodology for the assessment, nutritional diagnosis, intervention, monitoring and re-assessment, following the international evidence-based dietetics practice indications to ensure the patient receives safe, effective and high-quality nutritional care.

#### Psychologist

The aims of the psychologist intervention are the optimization of the patient’s awareness and acceptance of the disease, the identification and the promotion of changes in psychosocial and/or behavioral risk factors and the assessment (e.g. ACE III, FAB) or, if needed, a deeper investigation of the cognitive deficits through validated tests. Moreover, the intervention is focused on the provision of psychological support to the patient and their caregiver, promotion of medium and long-term treatment adherence and of disease management, activation of personal/socio familial resources.

### Data collection

**Fig 1:**
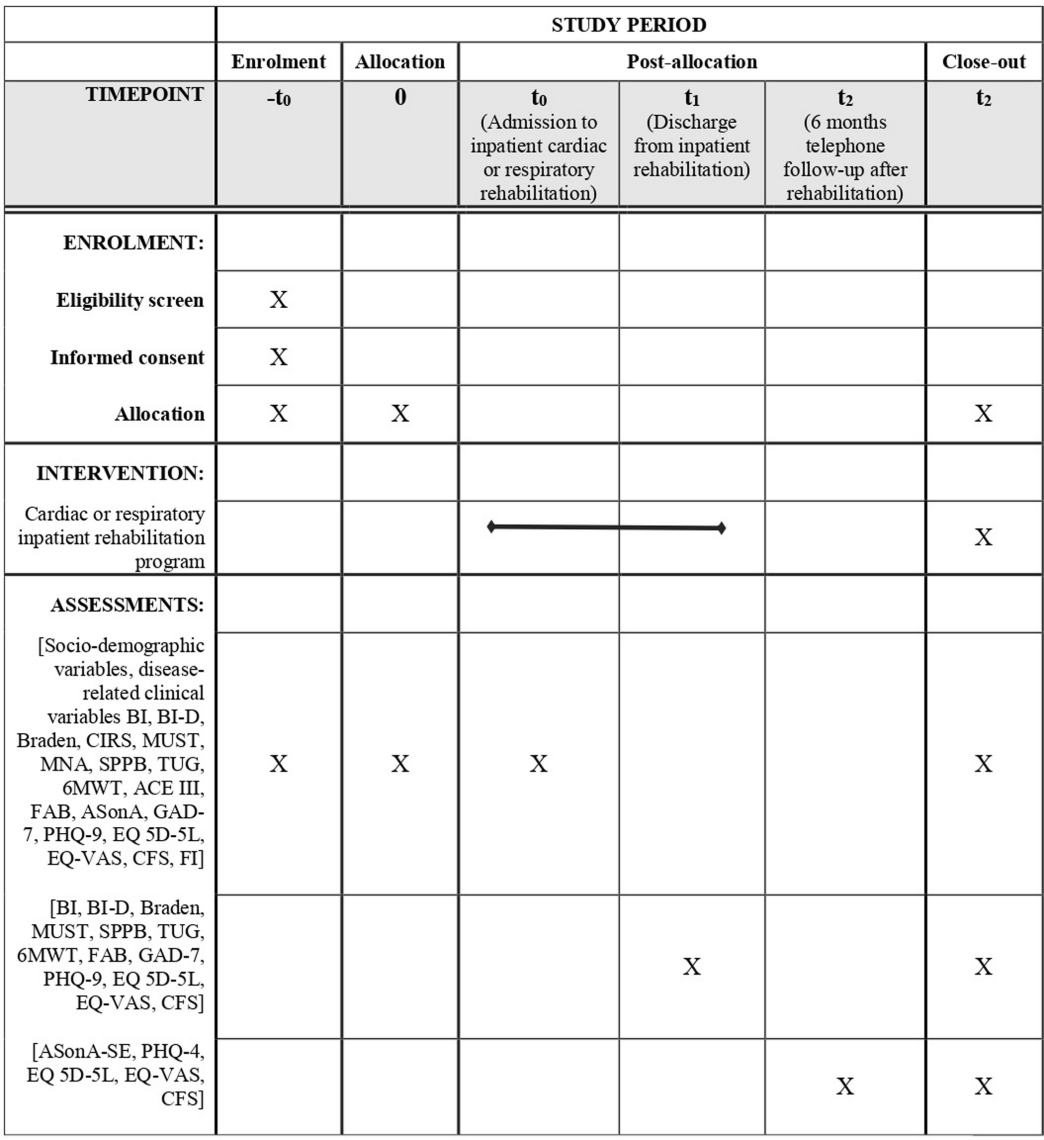
Schedule of enrollment, interventions, and assessments. BI, Barthel Index; BI-D, Barthel Index–Dyspnea; CIRS, Cumulative Illness Rating Scale; MUST, Malnutrition Universal Screening Tool; MNA, Mini Nutritional Assessment; SPPB, Short Physical Performance Battery; TUG, Timed Up and Go; 6MWT, 6 Minutes Walking Test; ACE III, Addenbrooke’s Cognitive Examination III; FAB, Frontal Assessment Battery; ASonA, Antecedents and Self-efficacy on Adherence Schedule; ASonA—SE, Antecedents and Self-efficacy on Adherence Schedule–Self-Efficacy; GAD-7, Generalized Anxiety Disorder– 7; PHQ-9, Patient Health Questionnaire– 9; PHQ-4, Patient Health Questionnaire– 4; EQ 5D-5L, EuroQol 5D 5l; EQ VAS, EuroQol Visual Analogue Scale; CFS, Clinical Frailty Scale; FI, Frailty Index.

The patients included in the study signed informed consent for all procedures and research explanations and underwent an evaluation at three different times that is at the baseline, at the hospital discharge, and at 6 months through a phone follow-up (Fig 1).

*Baseline (T*_*0*_*)*. The first assessment will be performed within a maximum of two to four days from the patient’s rehabilitation admission or, if necessary, after a therapeutic optimization, by a nurse (BI, BI-D, and Braden), by a physiotherapist (SPPB, TUG, and 6MWT), by a nutritionist (MUST and MNA) by a psychologist (ACE III, FAB, ASonA, GAD-7, PHQ-9, EQ 5D-5L, and EQ-VAS), while the cardiologist or pulmonologist will monitor individualized patient’s clinical pathway and performed the CIRS. Concerning frailty screening (CFS) and evaluation (FI) frailty, it will be performed by the three aforementioned healthcare professionals and nurses, from an interdisciplinary perspective.

*End of hospitalization (T*_*1*_*)*. After at least 2–3 weeks from the rehabilitation admission BI, BI-D, Braden, SPPB, TUG, 6MWT, MUST, FAB, EQ 5D-5L, EQ-VAS, and CFS will be re-administered as well as the collection of clinical indices related to the disease.

*6-months follow-up (T*_*2*_*)*. After six months from hospital discharge, the following scales will be administered during a telephone follow-up call: ASonA-SE, PHQ-4, EQ 5D-5L, EQ VAS, and CFS.

The study was approved by the Institutional Review Board and Central Ethics Committee of the ICS Maugeri SpA SB (CEC) (approval number: CEC N.2424, 23/04/2020), and it has been registered on January 28, 2022 with the ClinicalTrials.gov NCT05230927 registration number (clinicaltrials.gov/ct2/show/NCT05230927).

As to safety considerations, the only risk associated with participation concerns the physiotherapy evaluation (SPPB, TUG, and 6MWT), since there may be a risk of falls. To prevent this issue, patients must wear the correct clothes (e.g, closed-toe shoes to prevent sliding) and physiotherapists carefully follow the patients throughout the performances to intervene in case of need.

### Statistical analysis

#### Sample size

Cognitive impairment, anxiety, and depression are reported in about 20%-30% of patients from populations similar to ours [5, 8, 12, 14] while the prevalence of frailty is reported to be as high as up to 50% [21, 27]. Hence, to estimate the required sample size, we assumed an expected prevalence of 25%. Accordingly, the sample size required to estimate this prevalence with a 95% level of confidence and a margin of error of 5% was 288 subjects. Making a conservative choice, we assumed a sample size equal to 300 subjects. This number allows to estimate, with the same accuracy, prevalence values up to 50% with a 90% level of confidence.

#### Statistical analysis

Continuous variables will be expressed as mean ± standard deviation, while categorical variables will be expressed as a percentage.

The investigated variables, before and after intervention, will be assessed using paired sample t-tests or Wilcoxon signed-rank tests, depending on the sample distribution. Pearson correlation coefficient or Spearman rank correlation coefficient tests will be used to evaluate the associations between these variables. A general linear model will be applied to find the factors associated with clinical, functional, and frailty outcomes. Data will be analyzed using SAS/STAT statistical package, release 9.4 (SAS Institute Inc., Cary, NC, USA). The significant level will be set at p < 0.05.

## Discussion

The primary endpoint of the study is to define a multidimensional profile of the two clinical samples to better plan the rehabilitative intervention. The assessment of clinical and functional status, cognitive impairment, treatment adherence, psychosocial characteristics, and frailty could provide more specific and useful information about the main features to be considered in the evaluation and treatment of older patients suffering from CHF and COPD undergoing a rehabilitative pathway.

The secondary endpoint is to define the usability of the CFS with chronic patients in an interdisciplinary rehabilitation setting to routinely screen frailty. We expected that this tool results reliable in comparison to the gold standard (FI).

Furthermore, we expected to use the patients’ multidimensional profiles in order to tailor and improve the rehabilitative intervention over time and to implement the CFS scale to monitor the rehabilitative process.

### Strengths and limitations

A first limitation of this study concerns the estimated sample size since the COVID-19 pandemic outbreak could interfere with the recruitment of patients. A second limitation regards the assessment of treatment adherence, psychological distress and quality of life (QoL), evaluated through self-report measures. This limitation could be due to different reasons such as risk of false-positives and lack of sensitivity to change, psychometric adequacy of the assessment instrument and social desirability bias. Nevertheless, some advantages like high practicality of use, clinical and research applicability, and cost-effectiveness could be identified as positive aspects. Another strength of the study consists in the involvement of different healthcare professional figures that perform the evaluation and the intervention in a multidisciplinary way. Finally, frailty is often considered in rehabilitation settings, but its evaluation is not always performed with standardized methods and measures as in the present research protocol.

## Supporting information

S1 Appendix (DOC)

S2 Appendix (DOCX)
