# Supplementary material for: Cognitive impairment, frailty and rehabilitation outcome in older cardiorespiratory patients. DEC_FRAinRIAB: Study protocol
Source: PLoS One. 2022 Aug 4;17(8):e0272132. doi: 10.1371/journal.pone.0272132 (PMC9351997; doi:10.1371/journal.pone.0272132)
Supplement: S2 Appendix — (DOCX) [file pone.0272132.s002.docx]

**Cognitive impairment, frailty and rehabilitation outcome in elderly patients with cardiorespiratory disease. DEC_FRAinRIAB**

**Rationale**

A consistent number of studies in the last few years highlighted that the functional and clinical worsening in patients with cardiac and/or respiratory disease/s increase the risk of cognitive decline. The characteristics of this impairment are mainly related to dysfunctions in the frontal lobe and subcortical areas of the brain that led to executive functions and attention deficits, as well as memory dysfunctions (in particular in working memory and learning abilities) and in psychomotor speed (Alosco et al., 2014; Ampadu & Morley, 2015; Cameron, Gallagher, & Pressler, 2017; Cannon et al., 2017; Dodd, Getov, & Jones, 2010; Kakkera, Padala, Kodali, & Padala, 2018; Millan-Calenti et al., 2011). These deficits could appear in the Mild Cognitive Impairment (MCI) syndrome that is defined by a set of clinical, cognitive and functional criteria and it is characterized by a decline in cognitive performance over and above the normal expected variations due to age and educational level (Petersen et al., 1999).

The literature reports a greater diffusion of screening procedures for cognitive deficits in patients with cardiac diseases compared to patients with respiratory diseases. However, in both populations, the interest for cognitive impairment is justified by multiple reasons: the numerous exacerbations of the disease and re-hospitalizations, the difficulty in following complex therapeutic regimens and recognizing worsening of symptoms, the reduced functional autonomy and the rehabilitation outcome (Baird, Lovell, Johnson, Shiell, & Ibrahim, 2017; Currie, Rideout, Lindsay, & Harkness, 2015; Dulohery, Schroeder, & Benzo, 2015; Pierobon et al., 2018, 2017; Pressler, Kim, Riley, Ronis, & Gradus-Pizlo, 2010). Although recently the Italian Society of Geriatrics and Gerontology has raised the threshold for the definition of the 'elderly' patient from 65 to 75 years to better adapt to the current physical and mental performance of men and women living in economically developed countries and to the demographic situation of the Italian population (“QUANDO SI DIVENTA ‘ANZIANI’? | SIGG Società Italiana di Gerontologia e Geriatria,” n.d.), we considered in this study to adhere to the previous definition in order not to lose the MCI that characterizes the initial stages of cognitive decline (Langa & Levine, 2014). Therefore the three classes of 'elderly' patients that we will enroll will be defined as follows: “young old” (65–74 years), “old” (75–84 years), and "old-old" (≥85 years). In general, the age of the eligible sample is defined as ≥ 65 years.

It should be emphasized that the progressive aging of the population on the one hand and the improvement of surgical and therapeutic intervention techniques on the other, mean that compared to the past more elderly patients are suffering from heart disease, severe respiratory disease and/or from polypathologies. In this multifaceted framework of clinical situations, the complex management of therapies by patients led to an important risk that is the non-adherence with therapeutic prescriptions. The latter has serious consequences in terms of disability, hospitalizations, re-hospitalizations and a higher mortality rate (Sabaté, 2003). Furthermore, in chronic diseases, emotional factors, such as anxiety and depression, also play an important role in disease adaptation and in the rehabilitation outcome in both cardiac (Fattirolli et al., 2018; Sommaruga et al., 2018) and respiratory diseases (Fan & Meek, 2014; Fattirolli et al., 2018; Pierobon et al., 2017).

Alongside the problems relating to emotional aspects and cognitive decline, the frailty syndrome is noteworthy, particularly in the elderly and in the presence of cardiac/respiratory diseases. Frailty is associated with the loss of functionality that leads to greater vulnerability to adverse events such as the increased risk of falls, hospitalization, institutionalization, disability and mortality (L P Fried et al., 2001). Frailty is essentially defined by two paradigms. The biomedical paradigm (Linda P Fried, Ferrucci, Darer, Williamson, & Anderson, 2004) defines frailty as “a physiological syndrome characterized by the reduction of functional reserves and decreased resistance to stressors resulting from the cumulative decline of multiple physiological systems that cause vulnerability and adverse consequences”. At the operative level (L P Fried et al., 2001), physical frailty can be identified by the presence of three or more of the following components: reduction in body weight, fatigue, reduction in muscle strength, reduced physical activity and reduction in walking speed.

The bio-psycho-social paradigm (Gobbens, Luijkx, Wijnen-Sponselee, & Schols, 2010), on the other hand, defines frailty as "a dynamic state that affects an individual who experiences losses of one or more functional domains (physical, psychic, social), caused by the influence of several variables that increase the risk of adverse health outcomes”. This type of paradigm refers to a multidimensional assessment of the elderly, which includes, in addition to the assessment of the functional state, the psychological, cognitive, social, economic and spiritual profile. Therefore, in the frail elderly it is necessary the multidisciplinary evaluation of the functional (Instrumental Activities of Daily Living, IADL) and clinical status, the cognitive functions and psycho-affective status, the pharmacological treatment, the socio-economic situation and the individual needs, preferences and values (Consiglio Sanitario Regionale, 2003).

Frailty screening or assessment scales provide predictive information on the risk of death and institutionalization and they are a good predictor of acute hospital outcomes too (Abete et al., 2017; Strini & Schiavolin, 2019; Sze, Pellicori, Zhang, Weston, & Clark, 2019; Uchmanowicz et al., 2018). Instead, concerning what emerges from international literature, in rehabilitation cardiology, despite the increase in the presence of elderly patients, the clinical and prognostic relevance of frailty has not yet been well defined and measured (Vigorito et al., 2017). On the other hand, a recent study points out that frailty is present in 1/4 of outpatient COPD patients and, while being an independent predictor of rehabilitation program interruption, it is also easily reversible in the short term after rehabilitation (Maddocks et al., 2016). This result is also emphasized by Holland and colleagues who invite us to consider frailty as one of the relevant aspects in rehabilitative treatment (Holland, Harrison, & Brooks, 2016; Pandey et al., 2019).

**Aims:**

In light of the data in the literature, the purposes of this prospective observational study are to evaluate the following objectives:

1. At baseline, the presence of cognitive impairment, anxiety, depression, the assessment of self-reported adherence to therapeutic prescriptions and frailty in a sample of elderly patients (age ≥65) with chronic cardiorespiratory disease admitted for a cardiorespiratory rehabilitation cycle and the correlation with disease severity and functional aspects.
2. In follow-up, the impact that these factors have on the rehabilitation outcome at the end of hospitalization and on the state of health at six months (telephone interview).
3. A further objective is to verify the appropriateness of a screening scale for the measurement of frailty (CFS) (Rockwood et al., 2005) compared to a gold standard consisting of the "Frailty Index" (Abete et al., 2017) in the cardiorespiratory rehabilitation field

**Timeline:**

• (1^st^- 2^nd^ month) Materials acquisition and coordination

• (3^rd^- 14^th^ month) Patient enrollment and data acquisition at baseline and at the end of hospitalization

• (9^th^- 20^th^ month) telephonic follow-up (six months after discharge)

• (21^st^ month) data collection evaluation and meeting between investigators

• (16^th^ - 24^th^ month) Data analysis, drafting of works and dissemination of results

| **PHASES** | **YEARS** | **I year** | | | | | | | | | | | | **II year** | | | | | | | | | | | |
| --- | --- | --- | --- | --- | --- | --- | --- | --- | --- | --- | --- | --- | --- | --- | --- | --- | --- | --- | --- | --- | --- | --- | --- | --- | --- |
|  | **MONTHS** | **1** | **2** | **3** | **4** | **5** | **6** | **7** | **8** | **9** | **10** | **11** | **12** | **1** | **2** | **3** | **4** | **5** | **6** | **7** | **8** | **9** | **10** | **11** | **12** |
| Materials acquisition and coordination | |  |  |  |  |  |  |  |  |  |  |  |  |  |  |  |  |  |  |  |  |  |  |  |  |
| Baseline and discharge | |  |  |  |  |  |  |  |  |  |  |  |  |  |  |  |  |  |  |  |  |  |  |  |  |
| Follow-up (6 months) | |  |  |  |  |  |  |  |  |  |  |  |  |  |  |  |  |  |  |  |  |  |  |  |  |
| Data analysis, drafting of works and dissemination of results | |  |  |  |  |  |  |  |  |  |  |  |  |  |  |  |  |  |  |  |  |  |  |  |  |

Research diagram timeline

**MATERIAL AND METHODS**

All inpatients admitted to the Cardiac Rehabilitation Department and to the Pulmonary Rehabilitation Department of ICS Maugeri – Tradate (Varese) and Montescano (Pavia) will be evaluated to participate to the study.

**Inclusion criteria:**

• CHF was defined as: I) signs (e.g. elevated jugular venous pressure, pulmonary crackles and peripheral edema) and symptoms of HF [New York Heart Association (NYHA) functional class II-IV] in the presence of reduced ejection fraction (LVEF <40%); or II) signs and symptoms of HF (e.g. elevated brain natriuretic peptides and significant structural heart disease/diastolic dysfunction) with mid-range ejection fraction (LVEF 40–49%) or preserved (LVEF ≥50%).

• Patients were diagnosed according to Global initiative for chronic Obstructive Lung Disease (GOLD) criteria (Stage II–IV, C–D); they were in clinically stable condition (no exacerbations in the last 3 months) with optimized stable pharmacological therapy (inhalation therapy with long-acting anticholinergic and/or β2-agonists, inhaled corticosteroids when needed).

All the patients signed written informed consent form for this study.

**Exclusion criteria:**

• Severe clinical conditions (severe chronic inflammatory diseases, neoplasia, previous neurologic diseases).

• No Italian education, illiteracy or with relapse into illiteracy.

• Severe visuo-perceptive deficits.

• Lack of motivation or refusal to underwent to the evaluation.

• Severe psychiatric conditions

• Severe cognitive impairment

If serious neurological and / or psychiatric problems not previously reported in anamnesis are detected, the patient will be excluded from the study and specialist consultancy will be requested.

**Procedure**

All recruited patients will undergo to:

• Collection of the medical history (including the pharmacological prescriptions, with particular regard to the use of psychotropic drugs and hypno-inducing drugs) and physical examination. The use of psychotropic drugs and hypno-inducing drugs will not be considered an exclusion criteria but it will be considered in the data analysis.

• Evaluation of the main blood tests (CBC, creatinine, azotaemia, sodium, potassium, TSH, GOT, GPT, bilirubin, vitamin B12 and folate, vitamin D and prealbumin).

• ECG, chest x-ray, cardiac echocolordopplergraphy (where indicated).

• Optimization of medical therapy during rehabilitation hospitalization.

• Execution of the 6 minute walk test (6MWT), SPPB, TUG.

• Educational sessions.

• Physical training (cycle ergometer / treadmill, armergometer, breathing and strength exercises where indicated, calisthenics exercises).

• Psycho-social counselling.

• Metabolic evaluation with prescription of a personalized diet where indicated

• Administration if functioning scales, comorbidity and autonomy (Barthel motor and dyspnea -COPD-, MUST, CIRS, Braden).

The rehabilitation treatment is carried out according to the company diagnostic therapeutic path (PDTA) in accordance with the most recent national and international guidelines (Mezzani et al., 2012; Piepoli et al., 2016; Singh et al., 2019)

**Instruments**

Socio-anagraphic and clinical schedule investigates socio-anagraphic variables (e.g. age, gender, marital status, caregiver), comorbidities and pharmacological prescription, as well as some clinical data and risk factors (e.g. Body Mass Index -BMI and smoking habit).

Addenbrooke's Cognitive Examination III (ACE III) which is a 20 minutes screening test designed for the early detection of cognitive deterioration. ACE III is considered to be useful for screening degenerative dementias when shorter tests are inconclusive and its sub-scales can be useful to screen decline in specific areas of cognitive functioning. It analyzes five cognitive domains: Attention-Orientation, Memory, Verbal Fluency, Language and Visuospatial abilities (Pigliautile et al., 2019).

In this research, ACE III scores allow to divide sample in two subpopulations: patients affected by MCI (ACE III total score impaired or borderline, or impaired score in at least a ACE III subtest); patient not impaired (normal ACE III total score).

Frontal Assessment Battery (FAB) is a neuropsychological screening test used as assessment for executive functions. It is a quick and easy test to administer consisting of six sub-tests that analyse various aspects related to the functioning of the frontal lobes: conceptualization, mental flexibility, motor programming, sensitivity to interference, inhibitory control and environmental (Appollonio et al., 2005).

The EQ-5D-5L is a generic quality of life measurement tool. It investigates five dimensions: mobility, self-care, usual activities, pain/discomfort and anxiety/depression. Each item has five levels: 1, no problem; 2, slight problem; 3 moderate problem; 4 severe problem, 5 extreme problem. The second section consists in a Visual Analogue Scale (VAS) from 0 (Worst imaginable health state) to 100 (Best imaginable health state), where the subject is asked to indicate the level of self-perceived wellness (EQ 5D-5L. https://euroqol.org/).

Patient Health Questionnaire-9 (PHQ-9) is a scale currently used in general medicine to determine the diagnosis, severity and subsequent monitoring of depressive illnesses of the patient. The PHQ is characterized by two questions: the first, divided into 9 sub-items, has the aim to identify 9 depressive symptoms within the last 2 weeks (in accordance with DSM criteria). Patients indicate for each symptom whether, it has bothered them "not at all", "several days", "more than half the days", or "nearly every day" on a 4-point Likert scale. The final score has a range between 0-27. Scores are organized into clinical variability ranges according to a continuum of symptom severity (0-4 = Absent; 5-9 = Subthreshold depression; 10-14 = Mild major depression; 15-19 = Moderate major depression; ≥ 20 = Major depression severe) (Kroenke, Spitzer, Williams, & Löwe, 2010; R L Spitzer, Kroenke, & Williams, 1999).

Generalized Anxiety Disorder-7 (GAD) is a questionnaire built to measure the severity of anxiety symptoms in the previous two weeks. The questionnaire consists of 7 items, and similarly to the PHQ-9, it organizes the severity of the responses on a 4-point likert scale (0, not at all; 1, several days; 2, more than half of the days; 3, nearly every day). The range scores range from 0 to 21, where scores of 5, 10 and 15 are considered cut-offs for mild, moderate and severe anxiety, respectively (Kroenke et al., 2010; Robert L Spitzer, Kroenke, Williams, & Löwe, 2006).

The Antecedents and Self-efficacy on Adherence Schedule (ASonA) is a self- report schedule aimed to evaluate cognitive, behavioral, and emotional determinants of pharmacological and non-pharmacological adherence. It is a 21-item schedule scored on a 5-point Likert scale (0=not at all, 4=very much). The schedule comprises 3 subscales: Antecedents (ASonA-A), which include health condition and health-related limitations acceptance, social support and knowledge about health condition; Self- efficacy (ASonA-SE), exploring patients’ self-care strategies, and patients’ ability to adhere to medication assumption and to non-pharmacological recommendations (i.e., physical activity, diet, alcohol consumption, and smoking avoidance); and Affectivity (ASonA-Aff) which measures patients’ emotional aspects in relation to the health condition. In previous studies, similar schedules, belonging to the schedules group of ASonA, revealed to be sensible instruments showing strong correlations between antecedents and self- efficacy in relation to adherence (Zanatta F, Nissanova E, Świątoniowska-Lonc N, Pierobon A, Callegari G, Olmetti F, Felicetti G, Karniej P, Polański J, Giardini A, 2020).

Clinical Frailty Scale (CFS) is a scale developed in Canada, which is based on clinical judgment only with a score ranging from 1 to 9: 1) Very Fit; 2) Well; 3) Managing Well; 4) Vulnerable; 5) Mildly Frail 6) Moderately Frail 7) Severely frail 8) Very severely Frail 9) Terminally Ill (“Frailty in Older Adults - Early Identification and Management - Province of British Columbia,” n.d.; Rockwood et al., 2005). This scale considers clinical data on the subject's cognition, mobility, functional abilities and comorbidity, which can be collected through the medical history obtained from the patient, the caregiver and/or other health care providers (Rockwood et al., 2005). It is a quick and easy tool, which can be administered in a clinical setting and in a variety of care settings, as it requires no special equipment and is faster to use than other evaluation models that require performance testing (Strini & Schiavolin, 2019).

Frailty Index (FI) is a frailty measurement tool created through a geriatric assessment study including non-institutionalized patients. The variables taken into consideration are: mobility, muscle strength, comorbidities, cognitive deficits, mood, anthropometric indices, mini nutritional assessment and social support. It is made up of 40 items and 17 additional items relating to the Social Support Scale (Abete et al., 2017; Searle, Mitnitski, Gahbauer, Gill, & Rockwood, 2008).

Short Physical Performance Battery (SPPB) (Guralnik et al., 1994) is used to investigate the association between the physical performance and a self-assessed disability by the subjects recruited at a 6-year follow-up. It is also considered an indicator of functional frailty (Perracini et al., 2019).

SPPB evaluate the functional capacity of the lower limbs, consist of three different tests which have been assigned a partial score ranging from 0 to 4. The total score ranges from 0 (worst performance) to 12 (best performance). The 3 tests investigate: 1. Balance (the request is to maintain a static balance for 10 seconds: first with feet together (1 point); then in semitandem position (1 point); and finally in tandem position (2 points or 1 point if the subject maintains balance for more than 3 seconds). If the subject fails, i.e. moves his feet before 10 seconds or seeks support, the test is interrupted and the next session starts (walk). 2. Walk: time taken by the subject to travel a distance of 3 or 4 meters. The subject starts from a standstill, repeats the test twice and the shortest time corresponding to scores from 0 (unable to walk) to 4 is recorded. The subject is allowed to use walking aids. 3. Chair stand: ability to lift off the chair without using the upper limbs. The subject is initially asked to get up from the chair once to test the ability. If the interviewer and the subject himself deem the task safe, the test is completed with the execution of 5 lifts from the chair in the shortest possible time. Scores ranges from 0 to 4 depending on the execution time.

Timed Up and Go Test (TUG), is a simple test to measure a person's mobility level and requires static and dynamic balancing skills. It measures the time it takes a person to get up from a chair, walk ten feet, turn around, came back to the chair and sit down again. During the test, the person should wear shoes and use any mobility aids normally used (Podsiadlo & Richardson, 1991).

6-minute walking test (6MWT) is a self-limited test used to measure functional exercise abilities in people with HF, SCA and COPD. It is a test in which the person is asked to walk as fast as possible compatible with his clinical condition for a time of 6 minutes, measuring the meters travelled (“ATS statement: guidelines for the six-minute walk test.,” 2002).

**Data collection**

*Baseline*

The first assessment will be performed within a maximum of two to four days from the patient's rehabilitation admission or, if necessary, after a therapeutic optimization, by a psychologist (MMSE, ACE-R, FAB, PHQ- 9, GAD-7, ASonA EuroQol 5D and EuroQol VAS) by a physiotherapist (SPPB, 6MWT and TUG) while the cardiologist or pulmonologist will perform the collection of indices related to the pathology that are usually monitored during the clinical routine. Concerning frailty, screening (CFS) and evaluation (Frailty Index) will be performed by the three aforementioned healthcare professionals and nurses, from an interdisciplinary perspective. A neurological consultation may be requested in case of finding of cognitive impairment in the initial tests in order to exclude specific organic causes.

*End of hospitalization*

After at least 14 days from the rehabilitation admission, FAB, EuroQol 5D, EuroQol VAS, SPPB, 6MWT, TUG and CFS will be re-administered as well as the collection of clinical indices related to the disease.

*Follow-up (6 months after discharge)*

Patients will be contacted by telephone by a psychologist to assess the perceived quality of life (EuroQol 5D and EuroQol VAS - verbal) and, in the presence of a relative/caregiver, the frailty screening (CFS) will also be performed.

**DATA ANALYSIS**

*Sample Size*

The study design impose the enrollment of at least 300 subjects. This sample size makes it possible to estimate prevalence values between 20% and 30% less than a precision of 5% with a confidence level of 95%. The assumption of prevalence values between 20% and 30% is to be considered valid, in the target population studied, as expected values of cognitive impairment (Cameron, Gallagher, & Pressler, 2017; Dodd, Getov, & Jones, 2010), anxiety and depression (Alosco et al., 2014; Fattirolli et al., 2018). Concerning the expected percentage of frailty, literature reports values that can be as high as 50% (Strini & Schiavolin, 2019; Vigorito et al., 2017). However, it was decided to use conservative estimates in light of the researchers’ clinical experience. It should be noted that the proposed number is able to estimate, with the same accuracy, prevalence values of up to 50% with a confidence of 90%.

**References**

Abete, P., Basile, C., Bulli, G., Curcio, F., Liguori, I., Della-Morte, D., … Cacciatore, F. (2017). The Italian version of the “frailty index” based on deficits in health: a validation study. *Aging Clinical and Experimental Research*, *29*(5), 913–926. https://doi.org/10.1007/s40520-017-0793-9

Alosco, M. L., Spitznagel, M. B., Cohen, R., Raz, N., Sweet, L. H., Josephson, R., … Gunstad, J. (2014). Reduced cerebral perfusion predicts greater depressive symptoms and cognitive dysfunction at a 1-year follow-up in patients with heart failure. *International Journal of Geriatric Psychiatry*, *29*(4), 428–436. https://doi.org/10.1002/gps.4023

Ampadu, J., & Morley, J. E. (2015). Heart failure and cognitive dysfunction. *International Journal of Cardiology*, *178*, 12–23. https://doi.org/10.1016/j.ijcard.2014.10.087

Appollonio, I., Leone, M., Isella, V., Piamarta, F., Consoli, T., Villa, M. L., … Nichelli, P. (2005). The Frontal Assessment Battery (FAB): normative values in an Italian population sample. *Neurological Sciences : Official Journal of the Italian Neurological Society and of the Italian Society of Clinical Neurophysiology*, *26*(2), 108–116. https://doi.org/10.1007/s10072-005-0443-4

Baird, C., Lovell, J., Johnson, M., Shiell, K., & Ibrahim, J. E. (2017). The impact of cognitive impairment on self-management in chronic obstructive pulmonary disease: A systematic review. *Respiratory Medicine*, *129*, 130–139. https://doi.org/10.1016/j.rmed.2017.06.006

Cameron, J., Gallagher, R., & Pressler, S. J. (2017). Detecting and Managing Cognitive Impairment to Improve Engagement in Heart Failure Self-Care. *Current Heart Failure Reports*, *14*(1), 13–22. https://doi.org/10.1007/s11897-017-0317-0

Cannon, J. A., Moffitt, P., Perez-Moreno, A. C., Walters, M. R., Broomfield, N. M., McMurray, J. J. V, & Quinn, T. J. (2017). Cognitive Impairment and Heart Failure: Systematic Review and Meta- Analysis. *Journal of Cardiac Failure*, *23*(6), 464–475. https://doi.org/10.1016/j.cardfail.2017.04.007

Consiglio Sanitario Regionale. (2003). Fragilità nell’anziano. *Regione Toscana*, 1–48.

Currie, K., Rideout, A., Lindsay, G., & Harkness, K. (2015). The Association Between Mild Cognitive Impairment and Self-care in Adults With Chronic Heart Failure: A Systematic Review and Narrative Synthesis. *The Journal of Cardiovascular Nursing*, *30*(5), 382–393. https://doi.org/10.1097/JCN.0000000000000173

Dementia test - Brain and Mind Centre. (n.d.). Retrieved September 21, 2020, from https://www.sydney.edu.au/brain-mind/resources-for-clinicians/dementia-test.html#calculator

Dodd, J. W., Getov, S. V, & Jones, P. W. (2010). Cognitive function in COPD. *The European Respiratory Journal*, *35*(4), 913–922. https://doi.org/10.1183/09031936.00125109

Dulohery, M. M., Schroeder, D. R., & Benzo, R. P. (2015). Cognitive function and living situation in COPD: is there a relationship with self-management and quality of life? *International Journal of Chronic Obstructive Pulmonary Disease*, *10*, 1883–1889. https://doi.org/10.2147/COPD.S88035

EQ-5D. https://euroqol.org/. Accessed 20 Feb. 2020.

Fan, V. S., & Meek, P. M. (2014). Anxiety, depression, and cognitive impairment in patients with chronic respiratory disease. *Clinics in Chest Medicine*, *35*(2), 399–409. https://doi.org/10.1016/j.ccm.2014.02.012

Fattirolli, F., Bettinardi, O., Angelino, E., da Vico, L., Ferrari, M., Pierobon, A., … Piepoli, M. (2018). What constitutes the “Minimal Care” interventions of the nurse, physiotherapist, dietician and psychologist in Cardiovascular Rehabilitation and secondary prevention: A position paper from the Italian Association for Cardiovascular Prevention, Rehabilitat. *European Journal of Preventive Cardiology*, *25*(17), 1799–1810. https://doi.org/10.1177/2047487318789497

Folstein, M. F., Folstein, S. E., & McHugh, P. R. (1975). “Mini-mental state”. A practical method for grading the cognitive state of patients for the clinician. *Journal of Psychiatric Research*, *12*(3), 189–198.

Frailty in Older Adults - Early Identification and Management - Province of British Columbia. (n.d.). Retrieved November 30, 2020, from https://www2.gov.bc.ca/gov/content/health/practitioner-professional-resources/bc-guidelines/frailty

Fried, L P, Tangen, C. M., Walston, J., Newman, A. B., Hirsch, C., Gottdiener, J., … Group, C. H. S. C. R. (2001). Frailty in older adults: evidence for a phenotype. *The Journals of Gerontology. Series A, Biological Sciences and Medical Sciences*, *56*(3), M146–M156. https://doi.org/10.1093/gerona/56.3.m146

Fried, Linda P, Ferrucci, L., Darer, J., Williamson, J. D., & Anderson, G. (2004). Untangling the concepts of disability, frailty, and comorbidity: implications for improved targeting and care. *The Journals of Gerontology. Series A, Biological Sciences and Medical Sciences*, *59*(3), 255–263. https://doi.org/10.1093/gerona/59.3.m255

Gobbens, R. J. J., Luijkx, K. G., Wijnen-Sponselee, M. T., & Schols, J. M. G. A. (2010). In search of an integral conceptual definition of frailty: opinions of experts. *Journal of the American Medical Directors Association*, *11*(5), 338–343. https://doi.org/10.1016/j.jamda.2009.09.015

Guralnik, J. M., Simonsick, E. M., Ferrucci, L., Glynn, R. J., Berkman, L. F., Blazer, D. G., … Wallace, R. B. (1994). A short physical performance battery assessing lower extremity function: association with self-reported disability and prediction of mortality and nursing home admission. *Journal of Gerontology*, *49*(2), M85–M94. https://doi.org/10.1093/geronj/49.2.m85

Holland, A. E., Harrison, S. L., & Brooks, D. (2016). Multimorbidity, frailty and chronic obstructive pulmonary disease: Are the challenges for pulmonary rehabilitation in the name? *Chronic Respiratory Disease*, *13*(4), 372–382. https://doi.org/10.1177/1479972316670104

Kakkera, K., Padala, K. P., Kodali, M., & Padala, P. R. (2018). Association of chronic obstructive pulmonary disease with mild cognitive impairment and dementia. *Current Opinion in Pulmonary Medicine*, *24*(2), 173–178. https://doi.org/10.1097/MCP.0000000000000458

Kroenke, K., Spitzer, R. L., Williams, J. B. W., & Löwe, B. (2010). The Patient Health Questionnaire Somatic, Anxiety, and Depressive Symptom Scales: a systematic review. *General Hospital Psychiatry*, *32*(4), 345–359. https://doi.org/https://doi.org/10.1016/j.genhosppsych.2010.03.006

Langa, K. M., & Levine, D. A. (2014). The diagnosis and management of mild cognitive impairment: a clinical review. *JAMA*, *312*(23), 2551–2561. https://doi.org/10.1001/jama.2014.13806

Maddocks, M., Kon, S. S. C., Canavan, J. L., Jones, S. E., Nolan, C. M., Labey, A., … Man, W. D. C. (2016). Physical frailty and pulmonary rehabilitation in COPD: A prospective cohort study. *Thorax*, *71*(11), 988–995. https://doi.org/10.1136/thoraxjnl-2016-208460

Magni, E., Binetti, G., Bianchetti, A., Rozzini, R., & Trabucchi, M. (1996). Mini-Mental State Examination: a normative study in Italian elderly population. *European Journal of Neurology*, *3*(3), 198–202. https://doi.org/10.1111/j.1468-1331.1996.tb00423.x

Mesquita, R., Janssen, D. J. A., Wouters, E. F. M., Schols, J. M. G. A., Pitta, F., & Spruit, M. A. (2013). Within-Day Test-Retest Reliability of the Timed Up & Go Test in Patients With Advanced Chronic Organ Failure. *Archives of Physical Medicine and Rehabilitation*, *94*(11), 2131–2138. https://doi.org/https://doi.org/10.1016/j.apmr.2013.03.024

Mezzani, A., Hamm, L. F., Jones, A. M., McBride, P. E., Moholdt, T., Stone, J. A., … Williams, M. A. (2012). Aerobic exercise intensity assessment and prescription in cardiac rehabilitation: a joint position statement of the European Association for Cardiovascular Prevention and Rehabilitation, the American Association of Cardiovascular and Pulmonary Rehabilita. *Journal of Cardiopulmonary Rehabilitation and Prevention*, *32*(6), 327–350. https://doi.org/10.1097/HCR.0b013e3182757050

Millan-Calenti, J. C., Maseda, A., Rochette, S., Vazquez, G. A., Sanchez, A., & Lorenzo, T. (2011). Mental and psychological conditions, medical comorbidity and functional limitation: differential associations in older adults with cognitive impairment, depressive symptoms and co-existence of both. *International Journal of Geriatric Psychiatry*, *26*(10), 1071–1079. https://doi.org/10.1002/gps.2646

Mioshi, E., Dawson, K., Mitchell, J., Arnold, R., & Hodges, J. R. (2006). The Addenbrooke’s Cognitive Examination Revised (ACE-R): a brief cognitive test battery for dementia screening. *International Journal of Geriatric Psychiatry*, *21*(11), 1078–1085. https://doi.org/10.1002/gps.1610

Pandey, A., Kitzman, D., Whellan, D. J., Duncan, P. W., Mentz, R. J., Pastva, A. M., … Reeves, G. R. (2019). Frailty Among Older Decompensated Heart Failure Patients: Prevalence, Association With Patient-Centered Outcomes, and Efficient Detection Methods. *JACC: Heart Failure*, *7*(12), 1079–1088. https://doi.org/https://doi.org/10.1016/j.jchf.2019.10.003

Perracini, M. R., Mello, M., de Oliveira Máximo, R., Bilton, T. L., Ferriolli, E., Lustosa, L. P., & da Silva Alexandre, T. (2019). Diagnostic Accuracy of the Short Physical Performance Battery for Detecting Frailty in Older People. *Physical Therapy*, *100*(1), 90–98. https://doi.org/10.1093/ptj/pzz154

Petersen, R. C., Smith, G. E., Waring, S. C., Ivnik, R. J., Tangalos, E. G., & Kokmen, E. (1999). Mild cognitive impairment: clinical characterization and outcome. *Archives of Neurology*, *56*(3), 303–308. https://doi.org/10.1001/archneur.56.3.303

Piepoli, M. F., Hoes, A. W., Agewall, S., Albus, C., Brotons, C., Catapano, A. L., … Binno, S. (2016). 2016 European Guidelines on cardiovascular disease prevention in clinical practice: The Sixth Joint Task Force of the European Society of Cardiology and Other Societies on Cardiovascular Disease Prevention in Clinical Practice (constituted by representati. *European Heart Journal*, *37*(29), 2315–2381. https://doi.org/10.1093/eurheartj/ehw106

Pierobon, A., Ranzini, L., Torlaschi, V., Bottelli, E. S., Giardini, A., Bruschi, C., … Sommaruga, M. (2018). Screening for neuropsychological impairment in COPD patients undergoing rehabilitation. *PLoS ONE*, *13*(8), 1–13. https://doi.org/10.1371/journal.pone.0199736

Pierobon, A., Sini Bottelli, E., Ranzini, L., Bruschi, C., Maestri, R., Bertolotti, G., … Giardini, A. (2017). COPD patients’ self-reported adherence, psychosocial factors and mild cognitive impairment in pulmonary rehabilitation. *International Journal of Chronic Obstructive Pulmonary Disease*, *12*, 2059–2067. https://doi.org/10.2147/COPD.S133586

Pigliautile, M., Ricci, M., Mioshi, E., Ercolani, S., Mangialasche, F., Monastero, R., … Mecocci, P. (2011). Validation study of the Italian Addenbrooke’s Cognitive Examination Revised in a young-old and old-old population. *Dementia and Geriatric Cognitive Disorders*, *32*(5), 301–307. https://doi.org/10.1159/000334657

Podsiadlo, D., & Richardson, S. (1991). The Timed “Up & Go”: A Test of Basic Functional Mobility for Frail Elderly Persons. *Journal of the American Geriatrics Society*, *39*(2), 142–148. https://doi.org/https://doi.org/10.1111/j.1532-5415.1991.tb01616.x

Pressler, S. J., Kim, J., Riley, P., Ronis, D. L., & Gradus-Pizlo, I. (2010). Memory dysfunction, psychomotor slowing, and decreased executive function predict mortality in patients with heart failure and low ejection fraction. *Journal of Cardiac Failure*, *16*(9), 750–760. https://doi.org/10.1016/j.cardfail.2010.04.007

QUANDO SI DIVENTA “ANZIANI”? | SIGG Società Italiana di Gerontologia e Geriatria. (n.d.). Retrieved March 23, 2020, from https://www.sigg.it/news-geriatria/quando-si-diventa-anziani/

Rockwood, K., Song, X., MacKnight, C., Bergman, H., Hogan, D. B., McDowell, I., & Mitnitski, A. (2005). A global clinical measure of fitness and frailty in elderly people. *CMAJ : Canadian Medical Association Journal = Journal de l’Association Medicale Canadienne*, *173*(5), 489–495. https://doi.org/10.1503/cmaj.050051

Rosen, S. L., & Reuben, D. B. (2011). Geriatric Assessment Tools. *Mount Sinai Journal of Medicine: A Journal of Translational and Personalized Medicine*, *78*(4), 489–497. https://doi.org/10.1002/msj.20277

Sabaté, E. (2003). *Adherence to long-term therapies: evidence for action*. World Health Organization.

Searle, S. D., Mitnitski, A., Gahbauer, E. A., Gill, T. M., & Rockwood, K. (2008). A standard procedure for creating a frailty index. *BMC Geriatrics*, *8*(1), 24. https://doi.org/10.1186/1471-2318-8-24

Senda, M., Terada, S., Takenoshita, S., Hayashi, S., Yabe, M., Imai, N., … Yamada, N. (2020). Diagnostic utility of the Addenbrooke’s Cognitive Examination – III (ACE-III), Mini-ACE, Mini-Mental State Examination, Montreal Cognitive Assessment, and Hasegawa Dementia Scale-Revised for detecting mild cognitive impairment and dementia. *Psychogeriatrics*, *20*(2), 156–162. https://doi.org/https://doi.org/10.1111/psyg.12480

Singh, D., Agusti, A., Anzueto, A., Barnes, P. J., Bourbeau, J., Celli, B. R., … Vogelmeier, C. (2019). Global Strategy for the Diagnosis, Management, and Prevention of Chronic Obstructive Lung Disease: the GOLD science committee report 2019. *The European Respiratory Journal*, *53*(5), 1900164. https://doi.org/10.1183/13993003.00164-2019

Sommaruga, M., Angelino, E., Della Porta, P., Abatello, M., Baiardo, G., Balestroni, G., … Pierobon, A. (2018). Best practice in psychological activities in cardiovascular prevention and rehabilitation: Position Paper. *Monaldi Archives for Chest Disease = Archivio Monaldi per Le Malattie Del Torace*, *88*(2), 966. https://doi.org/10.4081/monaldi.2018.966

Spitzer, R L, Kroenke, K., & Williams, J. B. (1999). Validation and utility of a self-report version of PRIME-MD: the PHQ primary care study. Primary Care Evaluation of Mental Disorders. Patient Health Questionnaire. *JAMA*, *282*(18), 1737–1744. https://doi.org/10.1001/jama.282.18.1737

Spitzer, Robert L, Kroenke, K., Williams, J. B. W., & Löwe, B. (2006). A brief measure for assessing generalized anxiety disorder: the GAD-7. *Archives of Internal Medicine*, *166*(10), 1092–1097. https://doi.org/10.1001/archinte.166.10.1092

Strini, V., & Schiavolin, R. (2019). Scale di valutazione della fragilità nell’anziano: Una revisione della letteratura. *Assistenza Infermieristica e Ricerca*, *38*(2), 87–98. https://doi.org/10.1702/3169.31501

Sze, S., Pellicori, P., Zhang, J., Weston, J., & Clark, A. L. (2019). Identification of Frailty in Chronic Heart Failure. *JACC: Heart Failure*, *7*(4), 291–302. https://doi.org/10.1016/j.jchf.2018.11.017

Uchmanowicz, I., Kuśnierz, M., Wleklik, M., Jankowska-Polańska, B., Jaroch, J., & Łoboz-Grudzień, K. (2018). Frailty syndrome and rehospitalizations in elderly heart failure patients. *Aging Clinical and Experimental Research*, *30*(6), 617–623. https://doi.org/10.1007/s40520-017-0824-6

Vigorito, C., Abreu, A., Ambrosetti, M., Belardinelli, R., Corrà, U., Cupples, M., … Doherty, P. (2017). Frailty and cardiac rehabilitation: A call to action from the EAPC Cardiac Rehabilitation Section. *European Journal of Preventive Cardiology*, *24*(6), 577–590. https://doi.org/10.1177/2047487316682579

Wang, B.-R., Zheng, H.-F., Xu, C., Sun, Y., Zhang, Y., & Shi, J.-Q. (2019). Comparative diagnostic accuracy of ACE-III and MoCA for detecting mild cognitive impairment. *Neuropsychiatric Disease and Treatment*, *Volume 15*, 2647–2653. https://doi.org/10.2147/NDT.S212328

Zanatta F, Nissanova E, Świątoniowska-Lonc N, Pierobon A, Callegari G, Olmetti F, Felicetti G, Karniej P, Polański J, Giardini A, J.-P. B. (2020). Psychosocial Predictors of Self-Efficacy Related to Self-Reported Adherence in Older Chronic Patients Dealing with Hypertension: A European Study. *Patient Prefer Adherence*, *14*, 1709–1718. Retrieved from https://doi.org/10.2147/PPA.S258999
